# The relationship between mental state decoding and real-world social functioning – An experience sampling investigation

**DOI:** 10.1016/j.scog.2026.100419

**Published:** 2026-01-10

**Authors:** Ronja Christensen, Eva Velthorst, Anne-Kathrin Fett

**Affiliations:** a Department of Psychology and Neuroscience, School of Health and Medical Sciences, City St George's University of London, London, United Kingdom; b Queen Square Multiple Sclerosis Centre, Department of Neuroinflammation, UCL Queen Square Institute of Neurology, University College London, London, United Kingdom; c Department of Research, GGZ Noord-Holland-Noord, Heerhugowaard, the Netherlands; d King's College London, Institute of Psychiatry, Psychology and Neuroscience, Department of Psychosis Studies, De Crespigny Park, London, United Kingdom

**Keywords:** Reading the mind in the eyes (RMET), Theory of mind, Mental state decoding, Social cognition, Psychosis, Social functioning, Experience Sampling Method (ESM), Ecological Momentary Assessment

## Abstract

**Introduction:**

Social cognition, particularly Theory of Mind (ToM), is thought to play a crucial role in social functioning. Patients with psychosis often exhibit ToM deficits, but research findings on associations with social outcomes in daily life remain conflicting.

**Objectives:**

This study investigated the relationship between mental state decoding, a core aspect of ToM, measured by the Reading the Mind in the Eyes Test (RMET), and quantity and subjective quality of real-life social interactions in patients with psychosis, first-degree relatives, and controls.

**Methods:**

A 7-day Experience Sampling Method (ESM) design assessed the number and quality of real-life social interactions, including time spent alone vs. in social company, loneliness, feelings of social exclusion, preferences for company, being alone by choice, enjoyment of solitude, and perceived relationship quality in 27 patients with psychosis, 17 first-degree relatives, and 26 controls. All participants completed the RMET.

**Results:**

Patients scored lower on the RMET compared to both relatives (β = −0.13, *p* = .006) and controls (β = −0.19, *p* < .001), suggesting mental state decoding deficits in patients. Relatives did not differ from controls (*p* = .17), suggesting no association with familial risk. Across groups, lower RMET scores predicted feelings of social exclusion (β = −0.05, *p* = .02) but there were no significant associations between RMET performance and other aspects of real-life social functioning (all *p* > .7).

**Conclusions:**

Lower RMET performance was linked to greater feelings of social exclusion across groups but was unrelated to other indicators of real-life social functioning, including social emotions or frequency of social interactions. This finding is in line with other recent ESM studies, and highlights the importance of other, possibly more proximal factors for real-life social functioning.

## 1 Introduction

Poor social functioning, broadly defined as the inability to fulfil social, occupational, or self-care responsibilities (Mueser and Tarrier, 1998), is common in individuals with a psychotic disorder. Importantly, these problems precede the onset of the first psychotic episode and remain stable throughout the illness course (Velthorst et al., 2017). Social functioning difficulties can be explained in part by clinical symptoms and neurocognitive problems. Social cognition has been suggested to mediate this relationship (Addington et al., 2010; Halverson et al., 2019) and to offer unique predictive value for social functioning in patients (Cowman et al., 2021; Fett et al., 2011; Halverson et al., 2019). In particular, Theory of Mind (ToM), referring to the attribution of mental states such as knowledge, beliefs, emotions, and intentions to others (Frith and Frith, 2005; Premack and Woodruff, 1978), has been highlighted as a potentially important predictor of social functioning in psychosis (Fett et al., 2011; Lemmers-Jansen et al., 2023). ToM deficits are well-established across the psychosis continuum and have been proposed as a potential trait-marker of psychosis (Bora, 2020; Bora and Pantelis, 2013; Healey et al., 2016; Sprong et al., 2007; van Neerven et al., 2021). They appear mostly stable over the course of the illness (Ayesa-Arriola et al., 2016; Bora and Pantelis, 2013) and persist even during phases of remission (Bora et al., 2009; de Sales et al., 2023; Green et al., 2012). ToM is thought to be crucial for social interactions as it allows for the prediction and interpretation of subtle social cues and facilitates appropriate reactions in social situations (Green et al., 2019). Thus, it seems plausible that deficits in ToM may contribute to patients' social functioning difficulties.

While meta-analytical work suggests that ToM is linked to social functioning in individuals with psychotic disorders (Fett et al., 2011; Halverson et al., 2019; Thibaudeau et al., 2021), the findings of individual studies are heterogenous. Some evidence supports the link between ToM deficits and poor social functioning in the early stages of psychosis (Lindgren et al., 2018; Pennou et al., 2021; Sullivan et al., 2013; Ventura et al., 2015), as well as in more chronic phases (Brüne, 2005; Brüne et al., 2011; Cassetta and Goghari, 2014; Horton and Silverstein, 2008; Pijnenborg et al., 2009; Roncone et al., 2002; Zhu et al., 2007). However, other studies have found no association between ToM and social functioning in individuals with psychosis (Catalan et al., 2018; Langdon et al., 2014; Simons et al., 2016; Sullivan et al., 2014). Such inconsistencies may in part be owed to variation in measurements. Importantly, many studies relied on retrospective self-report questionnaires for social functioning that may be subject to retrospective recall bias and may not accurately depict real-life social functioning. Indeed, more recent meta-analytic work indicates stronger associations between ToM and performance-based (e.g., role-play) assessments of functioning compared with questionnaire and interview-based measurements (Lemmers-Jansen et al., 2023; Thibaudeau et al., 2021). Developments in digital health research, including smartphone-based experience sampling, have emphasised the value of assessing social functioning as it occurs in naturalistic contexts (Granholm et al., 2019; Myin-Germeys et al., 2018). One previous study used the Experience Sampling Method (ESM), a structured self-report diary technique, to explore the associations between ToM capacity and real-life social functioning in psychosis (Schneider et al., 2020). Interestingly, this study found no associations between performance on the Hinting Task (a measure of mental state reasoning) or the Picture Sequencing task (a measure of goal-directed action understanding and temporal-social sequencing) and the time spent alone, or appraisal ratings of the company in two samples, thus challenging the assumption that this specific aspect of daily-life social functioning depends on ToM skills.

Another potential explanation for the inconsistent findings is the heterogeneity of ToM tasks that were employed in previous studies, which may relate differently to social functioning. ToM can be divided into two distinct processes (1) cognitive ToM (mental state reasoning), and (2) affective ToM (mental state decoding) (Bora et al., 2006; Demirlek et al., 2023). Mental state reasoning refers to the ability to infer another person's intentions and goals based on a cognitive understanding of their mental state and knowledge. By comparison, mental state decoding relies on observable cues about social context, such as facial expressions or tone of voice to infer the mental state of others. Mental state decoding is often measured using the Reading the Mind in the Eyes Task (RMET) (Baron-Cohen et al., 2001), which assesses participants ability to recognise complex emotions and infer mental states based on visual representations of eye regions. While most studies investigating links between social functioning and ToM capacity have made use of mental state reasoning tasks, such as the Hinting Task (Corcoran et al., 1995), there is some evidence that mental state decoding may be a better predictor of social functioning in individuals with psychosis. For example, Bora et al. (2006) found that performance on a mental state decoding task (RMET) and a mental state reasoning task (Hinting Task) was both higher in patients with good social functional outcomes as compared to those with poor outcomes, yet only RMET performance significantly predicted social functioning. The authors proposed several possible explanations. They noted that the RMET may rely more on spontaneous and automatic emotion recognition than verbal and effortful reasoning and may thus better capture the intuitive processes involved in real-world social perception. They also highlighted the RMETs dependence on complex visual and affective cues, and reduced reliance on language processing and working memory, which are often impaired in psychosis. These qualities may make the RMET a more ecologically valid and sensitive measure of the socio-cognitive mechanisms that support everyday functioning. These findings were replicated by McGlade et al. (2008), who found that RMET performance predicted social functioning more strongly than Hinting Task performance, and that RMET performance explained unique variance in social function even after controlling for verbal IQ. More recently, Demirlek et al. (2023) linked mental state decoding, as measured with the RMET, to brain regions implicated in emotion processing, empathy, and social perception, including insular and superior temporal cortical measures, and white matter tracts. In contrast, mental state reasoning (Hinting Task) was linked to a different and only partial overlapping set of temporal and frontal regions involved in dynamic social information processing and higher order perspective-taking. These findings contribute to the overall argument that emotional mental state decoding contributes differently to social functioning than mental state reasoning.

It is important to note, however, that both Bora et al. (2006) and McGlade et al. (2008) made use of retrospective self-report or informant-based social functioning questionnaires, which measure social functioning largely based on participants' ability to engage in self-care, recreational or occupational activities. While these measures capture functional capacity, they do not assess the subjective experience of social interactions, such as perceived trust, inclusion, or satisfaction, which are crucial for understanding how individuals engage with and appraise their social world. In line with this, Achim et al. (2023) linked poorer ToM to reduced communication quality and less effective collaboration in interactive tasks among people with schizophrenia. Moreover, these questionnaire measures are subject to the aforementioned limitations of retrospective recall bias and informant bias and may not reflect momentary fluctuations in social emotions and engagement. Alternative assessments that have been employed by for example, Mehl et al. (2010) include role-play-based measures of social performance that provide a more immediate and observable measure of social skill. However, such structured, simulated settings may still not accurately depict qualitative and quantitative aspects of real-life social functioning, which also depend on spontaneous behaviour, contextual interpretation, and internal appraisals. Social performance tasks often emphasise social skills (i.e., the capacity to behave appropriately in social situations), while social functioning encompasses broader, real-life patterns of social participation and subjective incentive and appraisal hereof. Studies combining both quantitative (e.g., frequency or duration of interactions) and qualitative (e.g., perceived trust, exclusion, or loneliness) assessments of social interactions in real-life contexts are needed to elucidate the complex interplay between social behaviour and subjective experience, and to provide a more nuanced understanding of social functioning in psychosis.

In the current study, we aimed to explore the relationship between mental state decoding (as measured by the RMET) and real-life social interactions, specifically with regards to quantity and individual appraisals of the quality of daily social interactions, complementing Schneider et al., 2020 with additional measures of social cognition and aspects of real-life social functioning. We investigated this relationship in individuals with a non-affective psychotic disorder, healthy first-degree relatives, and controls.

Healthy first-degree relatives have an increased genetic vulnerability to psychosis, but do not present with clinical symptoms, and therefore confer an intermediate phenotype between patients and healthy controls. Therefore, they allow insight into whether mentalising difficulties and altered social functioning are markers of familial risk or specific to the psychotic state. Specifically, we wanted to investigate whether mental state decoding abilities could predict time spent alone, preference for being alone, and subjective appraisals of social interactions including feelings of trust, safety, loneliness, and acceptance. Indeed, subjective appraisal of both quality and quantity of social relationships may not necessarily be related to the actual quantity or frequency but must, by definition, be considered the primary constituent of social functioning. In the context of psychosis, individuals may experience social environments differently, even when their observable behaviour appears similar, and subjective appraisals may be more closely tied to internal cognitive-affective processes like mental state decoding.

We hypothesised that lower performance on the RMET would be associated with different aspects of real-life social functioning, specifically (i) more time spent alone, (ii) higher loneliness, (iii) greater feelings of social exclusion, (iv) stronger preference for being alone when with others (and lower preference for company when alone), (v) higher enjoyment of being alone, and (vi) lower perceived relationship quality.

## 2 Methods

### 2.1 Sample

The current sample was part of the DECOP study and comprised 70 participants across three groups: 27 patients with non-affective psychotic disorder, 17 healthy (never-psychotic) first-degree relatives and 26 healthy controls (not related to study patients); also previously reported in Hanssen et al. (2022) and Fett et al. (2022)). All participants were required to be aged between 18 and 65 and to have a sufficient command of English. Patients were enrolled if they met the ICD-10 criteria for a non-affective psychosis diagnosis (World Health Organization, 1992) and showed stability on their current pharmacological treatment for more than six weeks. Participants were excluded from the study if they had any history of neurological conditions or alcohol/drug dependence within 6 months of screening. Patients were recruited from the South London and Maudsley NHS Foundation Trust, the ‘Consent for Consent c4c’ initiative, the OXLEAS, NELFT and SEPT NHS Foundation Trust in cooperation with the Mental Health Research Network and through other studies within the Department of Psychosis Studies in the Institute of Psychiatry, Psychology and Neuroscience (IoPPN), King's College London. First-degree relatives were recruited through Mind and Rethink and were not related to the patients in the study. Healthy controls were recruited through recruitment advertisements within the IoPPN and online websites such as Gumtree and Callforparticipants. All study procedures were approved by the London-Harrow Research Ethics Committee (14/LO/0710) and followed the ethical standards of human experimentation as of the 1975 Helsinki Declaration and 2008 revisions.

### 2.2 Materials and methods

A demographic questionnaire was used to gather information on age, gender, ethnicity, living status and medication.

#### 2.2.1 Experience sampling method (ESM)

Participants used the ESM application which could be installed either on their personal iPhone or on an iPod provided by the study. The application prompted participants to complete a short questionnaire up to 10 times per day by beeping, in a pseudo-randomised fashion occurring between 08.00 am and 10.30 pm for 7 consecutive days. The ESM questionnaire comprised 30–34 questions depending on the participants' answer to the question “I am on my own”, which dictated the subsequent questions. If participants indicated that they were alone, they were directed to questions regarding solitary experiences; if not, they were asked question about social context and appraisal. In the current study, we focused analyses on 8 ESM-based outcomes, as follows: (1) 'time alone', assessed with dichotomised response to item ”I am on my own” (yes/no), (2) ‘loneliness’, assessed with the item “I feel lonely”, (3) ‘social exclusion’, assessed with the item “I feel that others exclude me”, (4 and 5) ‘preference for company’, assessed with the item “Right now, I would prefer to be with others/would rather be alone”, depending on social context (response to 'time alone'), (6) ‘alone by choice’ assessed with the item “Being alone is my choice”, and (7) 'enjoyment of solitude' assessed when participants were alone with the item “I enjoy being on my own”. We computed a variable (8) ‘perceived relationship quality’, which was based on an average of the 5 ESM items: “In this company…I feel accepted”, “I feel close to this person/these people”, “This person/these people are dependable, “I trust this person/ these people”, and “I like this person/these people”, based on principal component analysis (component loadings ranged from 0.82 to 0.92). ESM items (except time alone) were rated on a 7-point Likert scale ranging from *not at all* (1) to *very much* (2).

#### 2.2.2 Positive and negative syndrome scale (PANSS)

Clinical symptoms in the patient group were assessed with the PANSS (Kay et al., 1987), which measure positive (7-item), negative (7-item) and general (16-item) psychosis symptoms. Items are rated on a 7-point Likert scale ranging from *absent* (1) to *extreme* (7), contributing to a minimum score of 30 to a maximum of 210.

#### 2.2.3 Reading the mind in the eyes task (RMET)

A revised version of the RMET (Baron-Cohen et al., 1997, Baron-Cohen et al., 2001) measures the ability to recognise complex emotions and infer mental states based on visual presentation of eye expressions. In the revised version, participants are presented with 36 photographs depicting the eye-region of a person displaying a complex emotion (e.g., playfulness) and are asked to select the most appropriate emotional state among four options (e.g., playful, comforting, irritated, bored). Participants were provided with a definition list for all included emotional words. Performance is calculated as the number of correctly identified emotions (0–36).

#### 2.2.4 General cognitive ability

The Wechsler Abbreviated Scale of Intelligence (WASI) subtests on vocabulary and matrix reasoning (Wechsler, 1999) were used as estimate of general cognitive ability, to adjust analyses for the potential confounding effects on ToM performance.

### 2.3 Procedure

Participants were invited to the testing site at the IoPPN where detailed information about the study was shared and informed written consent obtained. All testing was conducted by trained MSc-level researchers. All participants completed a demographic questionnaire, RMET, and the WASI subtests, and patients were further assessed on the PANSS. Participants completed further tasks which are beyond the scope of the current analysis (Fett et al., 2022; Hanssen et al., 2022). All participants were instructed how to complete the ESM questionnaires on an app downloaded either to their own iPhones or on an iPod provided by the study team. Use of the app was demonstrated, and all participants were instructed to respond upon being prompted by a beep over the course of the following 7 days. After the 7-day testing period, participants returned to the IoPPN where they completed the remaining questionnaires and cognitive tasks. Patients also completed the PANSS. Upon completion of the study, participants were asked to return borrowed iPods and received a financial compensation for participation and reimbursement of travel costs.

### 2.4 Statistical analysis

All data was analysed with Stata version 17. Analysis of variance tests were used to explore group differences in age, RMET test scores and WASI vocabulary and matrix test scores. Pearson's chi-square tests were used to explore group differences in gender, ethnicity, living status, nationality, and education. To investigate the relationship between RMET performance and social functioning outcomes, multilevel mixed-effects linear regression models were employed. These models accounted for the repeated ESM measurements within participants, allowing for a more precise estimation of within-subject and between-subject effects. Group (patients, relatives, controls) and RMET performance were included as fixed effects. Interaction terms between group and RMET performance were initially tested but removed where non-significant at (*p* = .05). All models were adjusted for demographic covariates (age, sex and education), as well as estimated cognitive ability (WASI IQ) where appropriate.

## 3 Results

### 3.1 Sample characteristics

Sample characteristics are reported in Table 1. Groups differed significantly in gender composition (X^2^(2) = 8.18, *p* = .02), educational level (X^2^(10) = 22.51, *p* = .01, ethnicity X^2^(2) = 6.88, *p* = .03) and living status (X^2^(4) = 16.69, *p* = 0.002). Patients had a significantly lower estimated cognitive ability compared to controls (β = −18.02, t(67) = −4.99, *p* < .001, 95% CI (−25.22 to −10.81)), as did relatives (β = −11.01, t(67) = −2.69, *p* = .009, 95% CI (−19.19 to −2.83)).

**Table 1:** Sample characteristics.

	Controls (*N* = 26)	Relatives (*N* = 17)	Patients (*N* = 27)	*p*
Age, *M (SD)*	35.8 (8.29)	36.88 (13.72)	39.62 (9.56)	.39
Gender, male *n* (%)	17 (65.38%)^a^	6 (35.29%)^b^	21 (77.78%)^a^	**.02**
WASI, *M (SD)*				
Matrix	58.15 (9.67)	54.65 (7.57)	49.37 (10.15)	
Vocabulary	60.35 (6.47)	50.88 (14.28)	48.11 (11.08)	
Estimated IQ	116.42 (10.79^)a^	105.41 (16.19)^b^	98.41 (13.09)^c^	**< .01**
PANSS, *M (SD)*				
Positive	–	–	1.85 (0.61)	–
Negative	–	–	2.16 (0.86)	–
General	–	–	1.71 (0.37)	–
RMET, % correct, *M (SD)*	0.78 (0.09)^a^	0.73 (0.14)^a^	0.58 (0.17)^b^	**< .001**
Living Status, *n* (%)				
Alone	8 (30.77%)	4 (23.53%)	20 (74.07%)	
With family/partner	12 (46.15%)	10 (58.82%)	7 (25.93%)	
Other	6 (23.08%)	3 (17.65%)	0	
Beep count	51.15 (12.54)	42.24 (11.67)	48.48 (12.92)	
Education, *n* (%)				.**01**
None	0	1 (5.88)	3 (11.11)	
Primary	0	0	2 (7.41)	
Secondary	7 (26.92)	0	9 (33.33)	
College	6 (23.08)	5 (29.41)	8 (29.63)	
University	13 (50)	9 (52.94)	5 (18.52)	
Other	0	2 (11.76)	0	
Ethnicity, White *n* (%)	16 (61.54)^a^	8 (47.06)^b^	7 (25.93)^c^	**.03**

WASI: Wechsler Abbreviated Scale of Intelligence; PANSS: Positive and Negative Syndrome Scale; RMET: Reading the Mind in the Eyes Task. Values in bold show an overall group effect that is significant at *p* < .05. Different superscripts indicate group differences that are significant at *p* < .05.

Descriptive statistics and group comparisons for ESM are shown in Table 2. Intercorrelations between RMET, gender and ESM variables are shown in Table 3.

**Table 2:** Descriptives and group comparisons of ESM variables.

	Controls (N = 26)	Relatives (N = 17)	Patients (N = 27)	*p*
% time spent alone	57.21 (24.34)^a^	44.66 (21.91)^a^	73.38 (27.66)^b^	**.02**
Loneliness	**2.01 (1.29)**^a^	2.56 (1.53)^b^	**2.71 (1.59)**^b^	**.04**
Feeling socially excluded	1.86 (1.32)	2.15 (1.33)	2.47 (1.52)	.18
Enjoy being alone	**5.15 (1.21)**^a^	4.74 (1.76)^a, b^	**4.39 (1.79)**^b^	**.04**
Prefer company when alone	3.07 (1.57)^a^	3.36 (1.72)^a, b^	3.97 (1.77)^b^	.08
Prefer being alone when with others	2.24 (1.57)	2.68 (1.96)	2.35 (1.78)	.88
Alone by choice	5.01 (1.43)	4.69 (1.91)	4.50 (1.71)	.36
Relationship quality rating	5.58 (1.42)	5.25 (1.66)	5.68 (1.32)	.69

Note. Values in bold show an overall group effect that is significant at p < .05. Different superscripts indicate group differences that are significant at p < .05.

**Table 3:** Group-wise intercorrelations of RMET, gender and ESM variables (averaged).

	**RMET**	**Gender**	**% time spent alone**	**Loneliness**	**Feeling socially excluded**	**Prefer to be alone when with others**	**Enjoy being alone**	**Alone by choice**	**Prefer to be in company when alone**
**Patients**									
Gender (1 = male)	**−.26**								
% time spent alone	**−.18**	**.35**							
Loneliness	**−.31**	**.34**	**.17**						
Feeling socially excluded	**−.52**	.01	**.23**	**.53**					
Prefer to be alone when with others	.08	**−.19**	**.17**	**.17**	**.44**				
Enjoy being alone	.08	**−.24**	.03	−.03	**.12**	**.43**			
Alone by choice	.09	**−.16**	.22	−.06	.02	**.36**	**.70**		
Prefer to be in company when alone	**−.27**	**.30**	−.03	−.01	**.02**	**−.45**	**−.52**	**−.34**	
Relationship quality	**.01**	**−.40**	**−.41**	**−.15**	**−.24**	**−.30**	.09	.08	.01

**Relatives**									
Gender (1 = male)	**−.30**								
% time spent alone	**.44**	**.12**							
Loneliness	**.23**	.10	**.21**						
Feeling socially excluded	.08	−.10	−.08	**.69**					
Prefer to be alone when with others	**.26**	.02	**.28**	**.64**	**.59**				
Enjoy being alone	.12	**−.30**	.09	**−.45**	−.05	.12			
Alone by choice	**.21**	**−.30**	**.15**	**−.44**	−.05	.05	**.91**		
Prefer to be in company when alone	.01	**.15**	−.07	**.51**	.11	.01	**−.78**	**−.77**	
Relationship quality	−.08	−.04	**−.20**	**−.59**	**−.41**	**−.68**	**.15**	**.26**	**−.03**

**Controls**									
Gender (1 = male)	.01								
% time spent alone	**−.16**	**.44**							
Loneliness	**−.37**	.06	**.35**						
Feeling socially excluded	**−.21**	**.15**	**.36**	**.87**					
Prefer to be alone when with others	**−.19**	.003	.13	**.40**	**.51**				
Enjoy being alone	**−.10**	**−.24**	−.08	**−.41**	**−.48**	**−.12**			
Alone by choice	.02	**.17**	**.32**	**−.22**	**−.32**	**−.21**	**.59**		
Prefer to be in company when alone	−.06	−.03	**−.21**	**.40**	**.47**	**.48**	**−.54**	**−.56**	
Relationship quality	**.24**	.05	**−.43**	**−.19**	**−.27**	**−.59**	**−.22**	**−.10**	**.17**

Note. Significant at *p* < .05 in bold. Abbreviations RMET: Reading the Mind in the Eyes Task.

### 3.2 RMET performance

There was a significant difference between groups in RMET score (F(2,66) = 12.81, *p* < .001, with patients performing lower than both relatives ((β = −0.13, t(67) = −2.85, *p* = .006, 95% CI (−0.22 – -0.04) and controls (β = −0.19, t(67) = −5.01, *p* <. 001, 95% CI (−0.27 – -0.12). Relatives did not significantly differ from controls in RMET performance (β = −0.06, t(67) = −1.39, *p* = .17, 95% CI (−0.15–0.03), (see previous reports (Varela et al., 2021)).

### 3.3 Social functioning and RMET

Patients spent an average of 73.4% of their time alone, which was significantly more than relatives (44.7%, β = 28.72, t(67) = 3.69, *p* < .001, 95% CI (13.18–44.26) and controls (57.2%, β = 16.17, t(67) = 2.34, *p* *=* .02, 95% CI (2.37–29.96), who did not differ from each other. Patients were lonelier than controls (β = 0.66, *p* = .02, 95% CI [0.12–1.19]) but did not significantly differ from relatives (β = 0.16, *p* = .62, 95% CI [−0.48–0.82]), nor did relatives and controls differ from each other (as previously published for a larger sample of the DECOP project in Bell et al. (2023).

Groups did not significantly differ in feelings of social exclusion X^2^ (2) =3.45, *p* = .18, or in whether participants were alone by choice (X^2^(2) = 0.27, *p* = .88). There was a trend towards a group difference in preference for company when being alone (X^2^(2) = 5.07, *p* = .08). Patients preferred to be in company of others when alone significantly more than controls (β = 0.71, *p* = .03, 95% CI [0.07–1.35]) but did not differ from relatives (β = 0.57, *p* = .15, 95% CI [−0.19–1.33]). Relatives did not differ from controls (β = 0.14, *p* = .71, 95% CI [−0.61–0.89]). The groups did not differ in terms of their preference of being alone when with others (X^2^(2) = 0.27, *p* = .88). Groups differed significantly in whether they enjoyed spending time alone, X^2^(2) = 6.21, *p* = .04. Patients enjoyed being alone significantly less than controls (β = −0.79, *p* = .02, 95% CI [−1.48 – -0.11]) but did not differ from relatives (β = −0.05, *p* = .89, 95% CI [−0.87–0.76]). Relatives and controls did not significantly differ either (β = −0.74, *p* = .07, 95% CI [−1.54–0.06]). No group differences were seen in perceived relationship quality (X^2^ (2) = 0.75, *p* = .69).

#### 3.3.1 RMET and real-life social functioning

The interaction of RMET score and group on loneliness was significant (X^2^ (2) = 5.85, *p* = .05). In controls, lower RMET scores significantly predicted loneliness (β = −0.11, *p* = .03, 95% CI [−0.22 – -0.01]), but this was not the case in relatives (β = 0.05, *p* = .31, 95% CI [−0.05–0.16]), or patients (β = −0.04, *p* = .13, 95% CI [−0.09–0.01]). The interaction of RMET score and group on feelings of social exclusion was not significant (X^2^ (2) = 3.30, *p* = .19) and was removed from the model. RMET predicted feelings of social exclusion across groups (β = −0.05, *p* = .02, 95% CI [−0.11 – -0.01]), with higher RMET scores predicting less feelings of social exclusion. For all other analyses, no significant interactions between RMET and group were found, leading to their removal from the models (all *p* > .12). There were no significant association between RMET performance and any other indicators of real-life social functioning (all *p* > .17).

## 4 Discussion

This study investigated the relationship between mental state decoding (as measured by the RMET) and different aspects of the quantity and perceived quality of real-life social interactions in a group of patients with a psychosis, first-degree relatives of patients and a group of controls using experience sampling. Patients scored significantly lower on the RMET compared to both relatives and controls and showed more social isolation, loneliness and social exclusion. However, RMET performance did not predict most of the real-life social functioning indices. The exception were feelings of social exclusion which were significantly associated with lower RMET scores across groups, signifying non-specificity to psychosis. In addition, higher levels of loneliness were significantly associated with worse RMET performance in controls.

### 4.1 RMET performance between groups

As hypothesised and previously reported for an overlapping sample (Varela et al., 2021), patients performed significantly worse than both controls and relatives on the RMET task, but contrary to expectations, relatives and controls did not significantly differ from each other. This suggests that mental state decoding deficits are dependent on psychotic state rather than genetic or familial risk of psychosis. This contrasts with findings from de Achával et al. (2010) who found that first-degree relatives of patients scored lower on RMET compared to controls. Notably, they recruited first-degree relatives of the patients included, potentially meaning that patients and relatives shared socio-economic and genetic risk factors unrelated to psychosis contributing to a similar neuropsychological profile. The current study recruited non-familial first-degree relatives, where this limitation does not apply. Moreover, our findings are consistent with those of Pentaraki et al. (2012) who found that RMET impairments in first-degree relatives disappeared after controlling for IQ, suggesting that lower cognitive ability may account for apparent ToM deficits in this group.

### 4.2 Time spent alone and appraisals of social company

Patients spent significantly more time alone (73.4%) than first-degree relatives (44.7%) and controls (57.2%), who did not differ from each other (see also Fett et al. (2022)). Patients reported higher levels of loneliness compared to controls (Bell et al., 2023), in line with the well-established link between psychosis and loneliness (Lim et al., 2018; Michalska da Rocha et al., 2018). While relatives' levels of loneliness were intermediate, they did not significantly differ from either group in loneliness. Interestingly, patients had a lower appraisal of being alone and a stronger preference for being with others when alone compared to controls. This contrasts with assumptions that people with psychosis prefer being alone and highlights the possibility that their increased isolation may be experienced as aversive. This discrepancy between desired and actual social interaction (both in terms of quality and quantity) aligns with the definition of loneliness (Hawkley and Cacioppo, 2010). Relatives again showed an intermediate position, similar to both groups but not significantly different from either. Notably, there were no group differences in perceived relationship quality, feelings of social exclusion or whether being alone was by choice, contrasting with patients' reports of not enjoying being alone as much or rather wanting to be with others when alone.

### 4.3 The predictive value of RMET for social functioning

Our third hypothesis that lower RMET performance would be associated with reduced social interaction and more negative appraisals of social interaction and interaction partners was partially confirmed. Across groups, higher RMET scores predicted lower feelings of social exclusion. Conversely, across groups RMET scores were not significantly related to the quantity or perceptions of social interaction or the absence thereof (e.g., percentage of time spent alone, preference for being with others when alone, or being alone when with others, enjoyment of being alone, whether participants were alone by choice, and perceived relationship quality). This suggests that the capacity to interpret others' mental and emotional states does not influence frequency or absence of social interactions or the appraisal of being alone or with others, or of other people. Interestingly, a better mentalising capacity, as indicated by higher RMET scores, was associated with lower levels of loneliness in controls. However, this was not the case for patients or relatives, suggesting that other factors may be influencing feelings of loneliness in these groups or that feelings of loneliness might be less variable.

This study adds to the existing literature by assessing many different facets of real-life social functioning, which are not limited by retrospective recall biases, thus providing an ecological insight into quantitative and additional qualitative aspects of social interactions as they happen.

Our findings are in line with those reported by Schneider et al. (2020) and support the argument that ToM capacity may not be a leading contributor of daily life social functioning in psychosis patients. Our study employed a specific mental state decoding task, which appeared only relevant in predicting feelings of social exclusion. One possible explanation is that difficulties in accurately decoding others' emotional states may lead to misinterpretations of social cues, increasing the likelihood of perceiving exclusion even in neutral or ambiguous contexts. Importantly, this association was also seen in relatives and controls and thus was not specific to individuals with a psychotic illness.

Taken together, our findings suggest that social withdrawal in psychosis may reflect constraints or difficulties in engaging socially rather than a reduced desire for connection. Patients' stronger preference for company despite spending more time alone, highlights a potential disconnect between social motivation and actual social behaviour – especially since they did not report higher rating of being alone by choice. Although RMET performance did not predict most aspects of daily life social functioning, its association with perceived social exclusion can be interpreted in two ways. Individuals with lower mental state decoding ability may receive more social signals of exclusion, for example due to reduced communicative quality (Achim et al., 2023), or they may be more prone to perceive interactions as exclusionary an ambigous situations even when exclusion is not intended. A further consideration is the nature of the RMET itself. Because the task requires inferring mental states from minimal visual information, it targets very specific perceptual-emotional decoding processes rather than broader social cognitive abilities. These processes may be relevant primarily in situations where subtle social cues must be interpreted, which could explain why RMET performance only related to feelings of social exclusion and not the other aspects of social functioning in our study. Finally, the RMET has received methodological criticisms related to its reliance on vocabulary and confounding effects of verbal ability, and 19% of experts recommended discontinuation of its use in a recent survey (Ziermans et al., 2026), highlighting the need for investigations with additional social cognitive measures.

In conclusion, it seems that mentalising may be important for certain daily life social appraisals, while its influence is not disease-specific and does not extend to all areas of social functioning. The fact that this association was consistent across groups, suggests that the capacity to decode mental state may play a role in social appraisals more generally. While consistent with the findings of Schneider et al. (2020), our findings may not generalize to other tests of ToM. Future research that seeks to investigate the associations between social functioning and social cognition should utilize a variety of social cognitive tasks to identify which social cognitive aspects are most important for quantitative and qualitative aspects of social functioning.

## CRediT authorship contribution statement

**Ronja Christensen:** Writing – original draft, Formal analysis, Data curation. **Eva Velthorst:** Writing – review & editing, Conceptualization. **Anne-Kathrin Fett:** Writing – original draft, Supervision, Methodology, Funding acquisition, Formal analysis, Data curation, Conceptualization.

## Declaration of competing interest

AF and EV are editorial board members of Schizophrenia Research: Cognition.
